# Obituary

**Published:** 1845-05

**Authors:** 


### OBITUARY.

We regret to have to record the death of Professor Thomas Sewall, M. D., of Washington, D. C., which occurred on Monday, the 10th instant, in the fifty-ninth year of his age. Dr. Sewall was a native of Augusta, in the state of Maine. He graduated at the Medical School of Boston, and about the year 1820 removed to the City of Washington, where his talents and acquirements, with an upright deportment and great urbanity of manners, soon procured for him the respect and patronage of a large portion of the inhabitants. He was appointed Professor of Anatomy in the Medical School of that place on its first organization, and continued to discharge the duties of the Chair, with distinguished ability, until the time of his death. By the public prints we observe, that the members of the profession of the city in which he resided, and the students of the College to which he was attached, adopted suitable measures to express their grief for his loss. Beside these, his remains were followed to the grave by a large concourse of people, including a number of eminent statesmen, and the distinguished citizens of the place.

